# Bioenergetic impedance: a network physiology framework for stress adaptation, maladaptation, and recovery

**DOI:** 10.3389/fnetp.2026.1967461

**Published:** 2026-09-29

**Authors:** Torsak Tippairote, Pruettithada Hoonkaew

**Affiliations:** 1 Department of Life and Consumer Sciences, University of South Africa, Pretoria, South Africa; 2 HP Medical Center, Bangkok, Thailand; 3 Department of Diagnostic Imaging, Mahachai Hospital, Samut Sakhon, Thailand

**Keywords:** bioenergetic impedance, network physiology, physiological resilience, recovery dynamics, stress adaptation

## Abstract

Stress adaptation depends not only on challenge magnitude but on the capacity of physiological systems to conduct, transform, buffer, and recover energetic demands. We propose *bioenergetic impedance* as a Network Physiology construct describing the time-dependent constraint on energetic throughput and recovery that arises when energetic pressure exceeds available biological capacity. Resistance, delay, congestion, and buffering burden may reflect mechanisms or manifestations of this mismatch, whereas compensatory rerouting may represent an adaptive response to constrained throughput. At the cellular level, substrate congestion and redox backpressure provide plausible mechanistic anchors, while metabolic rerouting and mitochondrial stress signaling may represent compensatory or signaling responses through which local energetic constraints may be accommodated or communicated. Their physiological consequence depends critically on reversibility. In *adaptive impedance cycling*, challenges that transiently exceed available capacity produce impedance followed by recovery, restoration or adaptive recalibration of flexible cross-system organization, and potentially expanded reserve. In *impedance lock-in*, repeated or unresolved challenge produces incomplete recovery, persistent residual constraint, compensatory allocation, reduced network flexibility, and constrained adaptive reserve. The framework therefore places recovery dynamics at the center of resilience and generates a testable prediction: adaptive states should show temporary, context-appropriate reconfiguration of cross-system interactions followed by restoration of flexible network organization, whereas maladaptive states should show incomplete recovery and persistent or functionally inappropriate network reconfiguration. Bioenergetic impedance thus provides a systems-level framework linking cellular energetic constraints with stress adaptation, maladaptation, and recovery across physiological networks.

## 1 Introduction: stress responses as energy transformation under constraint

Stress responses involve coordinated neuroendocrine, autonomic, immune, vascular, metabolic, and mitochondrial adjustments that mobilize and redistribute energetic resources in response to changing demand. From homeostasis and the general adaptation syndrome to allostasis, stress has therefore been understood as an active multisystem process that preserves stability through physiological change (Cannon, 1932; McEwen, 1998; Selye, 1950; Sterling and Eyer, 1988). Mitochondria have more recently been positioned as central integrators of stress signaling and energetic adaptation across biological levels (Picard et al., 2018; Picard and McEwen, 2018).

What remains less developed is a Network Physiology explanation for why coordinated stress responses resolve adaptively in some contexts yet become persistent and maladaptive in others. Adaptation requires not only mobilization of energetic resources but sufficient capacity to conduct, transform, buffer, and replenish them during recovery. Healthy adaptation therefore depends on completion of a respond–adapt–recover cycle that restores physiological flexibility and reserve for subsequent challenges (Tippairote et al., 2025; Tippairote et al., 2026).

Here, we propose bioenergetic impedance as a systems-level organizing construct describing how time-dependent energetic constraints and recovery dynamics shape stress responses across interacting physiological systems. The term is not intended to replace established metabolic, mitochondrial, vascular, inflammatory, or neuroendocrine mechanisms, but to organize their relationship to energetic throughput and recovery. Building on energetic models of stress, the *Energy Resistance Principle (ERP)*, and the *Energy Allocation System (EAS),* the framework asks how heterogeneous energetic demands encounter scale-specific constraints in biological throughput and recovery, how unresolved constraints propagate through physiological networks, and whether they resolve through recovery or persist as maladaptive network organization (Picard and Murugan, 2025; Schuler et al., 2026).

## 2 Bioenergetic impedance: from energetic resistance to network constraint

We propose that diverse stressors impose energetic demands that support defense, substrate mobilization, repair, and adaptation. We define *bioenergetic impedance* as the time-dependent constraint on energetic throughput and recovery that arises when energetic pressure exceeds available throughput and recovery capacity. Resistance, delay, congestion, and buffering burden may contribute to or manifest this state, whereas rerouting may represent a compensatory response. Recovery is the time- and resource-dependent process through which the constraint resolves; incomplete recovery may leave residual constraint that increases impedance during subsequent challenges (Behnke et al., 2026; Picard and Murugan, 2025; Tippairote et al., 2026).

### 2.1 Construct boundaries

Bioenergetic impedance has three important boundaries. *First*, it is scale dependent rather than a single global biological quantity: the organizing principle—energetic pressure exceeding available throughput and recovery capacity—may apply across cells, tissues, organs, and physiological networks, but mechanisms and measures differ by scale. *Second*, its physics-inspired terminology is heuristic; the framework implies neither electrical equivalence, common governing equations, nor a universal impedance metric. *Third*, impedance is not synonymous with mitochondrial dysfunction, inflammation, metabolic rerouting, lactate accumulation, or any individual mechanism or biomarker. Such processes are relevant only when they contribute to, reflect, or sustain a demonstrable demand–throughput–recovery constraint. The construct therefore organizes relationships among established biological processes rather than renaming them.

These processes also occupy different causal positions: limitations in delivery, transport, substrate handling, or oxidative capacity may contribute to impedance; respiratory limitation or redox backpressure may manifest constrained throughput; metabolic rerouting and stress signaling may represent compensatory or signaling responses; and outcome depends on whether recovery resolves the constraint and replenishes reserve.

For empirical testing, impedance and recovery should be operationalized separately. Impedance could be identified prospectively from the relationship between imposed demand and independently measured throughput or reserve, whereas recovery kinetics, residual deviation, or hysteresis would test whether the constrained state subsequently resolves. The principal terms are summarized in Box 1.

**BOX 1:** Physics-inspired relational grammar and associated biological terms.

Term	Operational meaning within the framework
Pressure	Energetic or adaptive demand imposed during challenge
Conductance	Capacity to support energetic throughput under a given demand
Flow/throughput	Realized processing and transformation of energetic resources into useful biological work; flow is heuristic shorthand
Resistance	A bottleneck limiting conductance or throughput; it may contribute to impedance but is not synonymous with it
Buffering capacity/capacitance	Capacity to temporarily accommodate demand–throughput mismatch; capacitance is its physics-inspired analogue. Buffering may draw on but is not equivalent to reserve
Reserve	Latent capacity recruitable during challenge, adaptation, or recovery
Recovery capacity	Capacity supporting resolution, repair, replenishment, and adaptive remodeling
Recovery	Time- and resource-dependent process through which impedance resolves and subsequent adaptive capacity is restored, recalibrated, or expanded

Taken together, these terms describe a relational sequence: energetic demand generates pressure, conductance governs the throughput achievable under that pressure, and buffering temporarily accommodates mismatch when demand exceeds realized throughput. Reserve represents latent capacity recruitable during challenge, whereas recovery capacity specifically supports resolution, repair, replenishment, and adaptive remodeling after challenge.

Bioenergetic impedance extends rather than replaces ERP, which describes energetic resistance when potential exceeds flux capacity, and complements EAS, which emphasizes neuroendocrine governance and prioritization of energetic resources (Picard and Murugan, 2025; Schuler et al., 2026). Bioenergetic impedance instead emphasizes energetic execution and recovery: whether allocated demands can be accommodated through scale-specific throughput and recovery capacities. Although analytically distinct, allocation and execution are bidirectionally coupled.

From a Network Physiology perspective, unresolved local constraints may alter signaling and resource allocation across interacting systems; whether such reconfiguration resolves during recovery becomes central to adaptive versus maladaptive trajectories (Figure 1).

**FIGURE 1:**
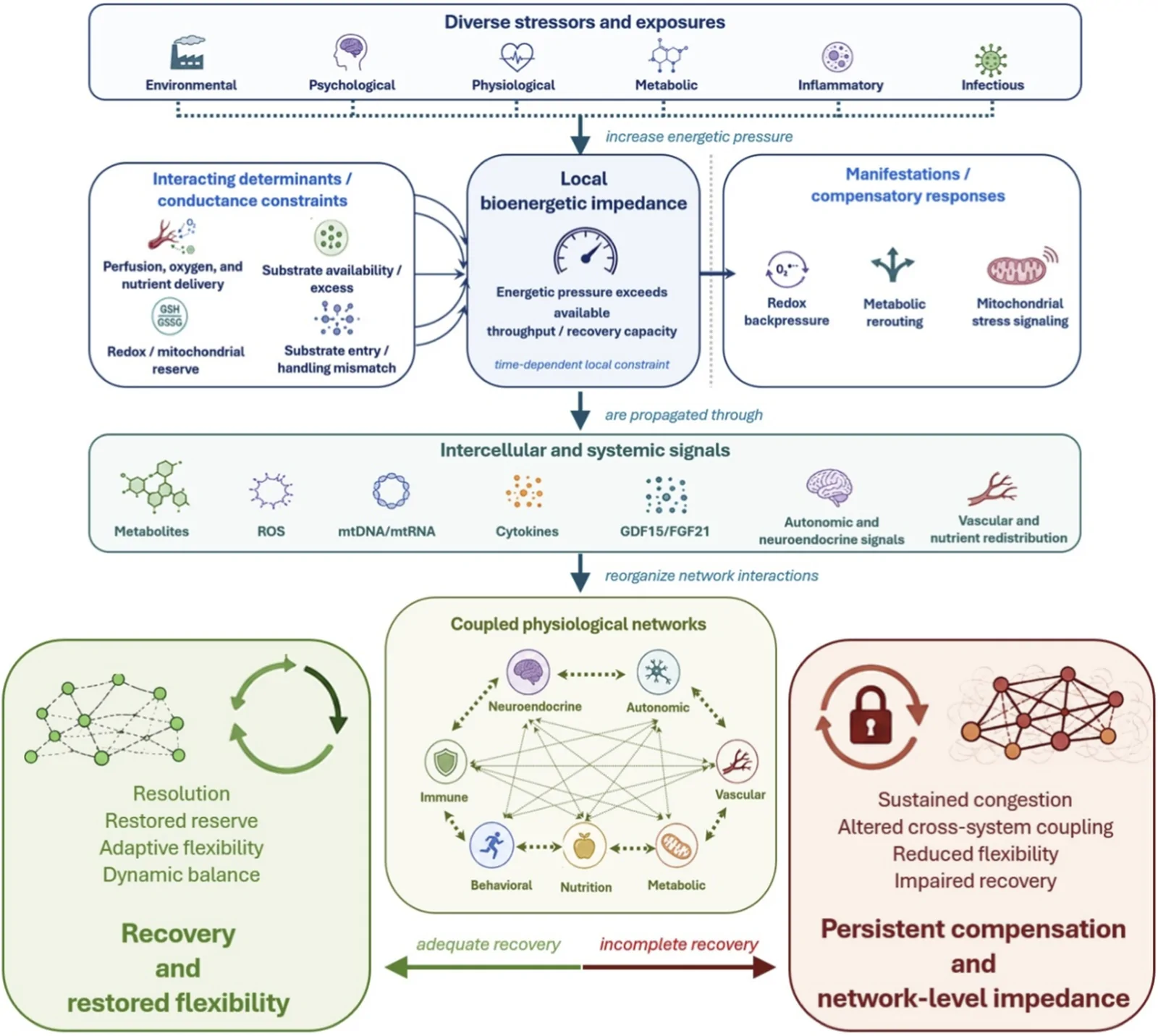
Multilevel architecture of bioenergetic impedance across the stress response. Diverse stressors increase energetic pressure on the organism. At the local level, interacting determinants—including perfusion, oxygen and nutrient delivery, substrate availability and handling, and redox or mitochondrial reserve—may constrain energetic throughput and recovery; these determinants may operate in parallel and are not intended as an obligatory sequence. Bioenergetic impedance denotes the time-dependent constraint that arises when energetic pressure exceeds available throughput and recovery capacity. In Figure 1, only the central node represents bioenergetic impedance itself; the surrounding components represent determinants, manifestations, or compensatory responses rather than forms of impedance. Redox backpressure may manifest constrained throughput, whereas metabolic rerouting and mitochondrial stress signaling may represent compensatory or signaling responses. Signals and resource reallocations generated by unresolved local constraints—including metabolites, ROS, mitochondrial nucleic acids, cytokines, mitokines, autonomic and neuroendocrine signaling, vascular responses, and nutrient redistribution—may alter interacting tissues and reorganize physiological networks. Sufficient recovery resolves impedance and restores or strengthens flexible network regulation; incomplete recovery may sustain distributed constraints, compensatory reconfiguration, and network-level impedance. The categories shown occupy distinct causal positions and represent scale-specific expressions of a common organizing principle rather than identical mechanisms or a universal impedance metric. Arrows indicate predominant, potentially bidirectional relationships rather than a strictly linear causal sequence.

## 3 Cellular origins of bioenergetic impedance

At the cellular level, bioenergetic impedance may arise when substrate supply, reducing-equivalent generation, or adaptive demand exceeds the capacity for oxidative flux, redox regulation, and recovery. Initiating constraints may arise from substrate excess, hypoxia, toxicants, infection, or psychosocial demand. The common feature is therefore not low ATP production or intrinsic mitochondrial dysfunction alone, but a mismatch between energetic demand and the cellular capacity to convert available resources into useful biological work (Muoio, 2014; Picard and Murugan, 2025; Picard and Shirihai, 2022; Tippairote et al., 2022).

The processes below are therefore presented as mechanistic anchors or compatible illustrations, not as interchangeable definitions or direct validation of the higher-order construct.

The first mechanistic anchor is substrate congestion and redox backpressure. When carbon and reducing equivalents enter metabolism faster than mitochondria can oxidize them, NADH and upstream intermediates accumulate and oxidative flux becomes constrained (Muoio, 2014; Wang and Patti, 2023). Mitochondrial throughput also depends on efficient import, folding, and assembly of predominantly nuclear-encoded proteins; constraints within these pathways may limit renewal of respiratory machinery and contribute to local conductance limitation (Song et al., 2021). Cells may respond by increasing respiratory throughput, restricting substrate entry, buffering excess pressure, or rerouting carbon and reducing equivalents. Insulin resistance during chronic nutrient excess illustrates a potentially adaptive restriction of substrate entry, but is compatible with the framework rather than direct evidence of bioenergetic impedance (Prentki et al., 2026).

A second cellular response is adaptive metabolic rerouting. Lactate production can regenerate NAD^+^ and sustain glycolytic flux when oxidative metabolism cannot fully accommodate glycolytic carbon and reducing-equivalent generation (Liberti and Locasale, 2016; Luengo et al., 2021). Such rerouting is not inherently pathological, and persistence alone does not indicate maladaptation or impedance. Its significance depends on whether it accompanies sustained demand–throughput mismatch and failure to restore oxidative flexibility, recovery capacity, or subsequent challenge tolerance.

A third response is mitochondrial stress signaling. Mitochondrial remodeling, integrated stress responses, ROS signaling, TCA-cycle metabolites, and mitochondrial nucleic-acid release can translate energetic strain into transcriptional, inflammatory, and immune signaling (Monzel et al., 2023; Picard and Shirihai, 2022; Ryan and Zanoni, 2026; West and Shadel, 2017). These responses provide plausible routes through which local energetic constraints may influence broader physiological networks.

Together, these mechanisms provide biologically plausible routes from local energetic constraint toward tissue and network reorganization, but their presence alone does not establish bioenergetic impedance.

## 4 From local bioenergetic constraints to physiological network reorganization

Because physiological systems are coupled, local bioenergetic constraints may alter signaling, resource distribution, or functional demand at other biological levels. A useful distinction can therefore be made among local impedance, propagation, and network-level impedance. *Local impedance* occurs when energetic pressure within a cell or tissue exceeds its throughput and recovery capacity. *Propagation* refers to signals, resource reallocations, or altered demands generated by unresolved local constraints through metabolites, cytokines, mitokines, autonomic and neuroendocrine signaling, vascular responses, or nutrient redistribution (Ryan and Zanoni, 2026). Propagation therefore does not imply transmission of a single mechanism; mechanistically distinct constraints may emerge in interacting tissues. *Network-level impedance* denotes the higher-order state in which distributed constraints and compensatory responses reorganize interactions among physiological systems and sustain a compensatory configuration (Bartsch et al., 2015; Burtscher et al., 2023; Ivanov, 2021).

This relationship is bidirectional rather than a one-way progression from cellular dysfunction to systemic consequences. Whole-organism neuroendocrine, immune, vascular, metabolic, and behavioral responses can alter the demand imposed on local energetic execution, while constraints arising during cellular execution can generate signals and resource reallocations that modify those same physiological networks.

Mitokines illustrate such propagation. In Metabolic Dysfunction-Associated Steatohepatitis (MASH), GDF15-mediated brainstem signaling suppressed hepatic inflammation independently of weight loss, providing an example of systemic stress communication compatible with the proposed architecture but not direct evidence of bioenergetic impedance or network lock-in (Wang et al., 2026).

From a Network Physiology perspective, propagation becomes important when it changes the organization of cross-system interactions rather than simply affecting more organs. In adaptive states, such reconfiguration should remain context appropriate and reversible; unresolved impedance may instead sustain compensatory organization across systems (Behnke et al., 2026). Network-level impedance is therefore proposed as a change in the timing, direction, strength, functional appropriateness, flexibility, and reversibility of physiological interactions rather than the sum of independent organ dysfunctions. Whether this reorganization resolves during recovery or persists with incomplete recovery distinguishes the adaptive and maladaptive trajectories developed in Section 5.

## 5 Adaptive and maladaptive trajectories: impedance cycling and lock-in

When challenge produces bioenergetic impedance, the distinction between adaptive and maladaptive trajectories depends primarily on whether that constraint subsequently resolves. During acute stress, impedance may arise transiently when energetic demand exceeds available throughput or recovery capacity. Transient impedance is not necessarily a failure state; its physiological consequence depends on reversibility: whether compensatory reconfiguration resolves and flexible, context-appropriate regulation is restored or adaptively recalibrated once the initiating exposure or trigger has resolved or been contained (McEwen and Wingfield, 2003; Picard et al., 2018). Resistance is therefore state dependent; responses that initially constrain harmful energetic flux may become maladaptive when the underlying pressure persists (Prentki et al., 2026).

In adaptive trajectories, transient impedance is followed by a recovery phase that completes the respond–adapt–recover cycle. Recovery may restore the system toward its individualized functional range, recalibrate it to a modified but functionally appropriate state, or, when adaptive remodeling expands capacity, produce hormetic expansion of reserve. Consistent with hormetic conditioning, the framework predicts that adaptive remodeling may produce smaller impedance excursions, faster resolution, and preserved or expanded reserve during comparable subsequent challenges (Figure 2A) (Calabrese and Mattson, 2024; Mattson, 2025; Picard et al., 2018; Tippairote et al., 2026).

**FIGURE 2:**
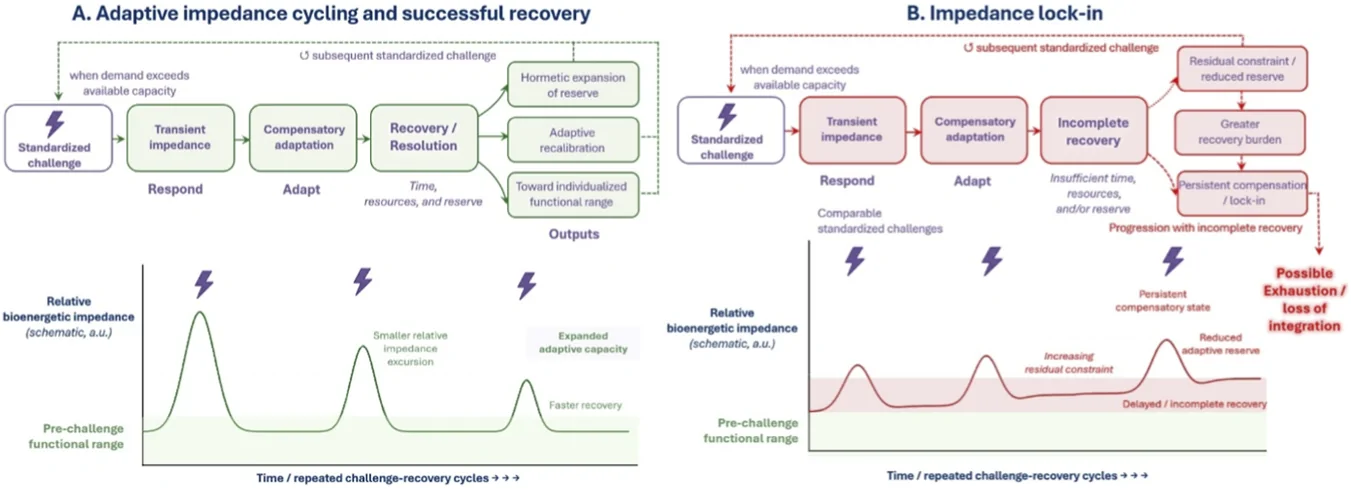
Adaptive impedance cycling and successful recovery versus impedance lock-in. Conceptual trajectories show relative bioenergetic impedance under repeated comparable standardized challenges; curves are illustrative and expressed in arbitrary units. **(A)** Adaptive impedance cycling. A challenge that transiently exceeds available capacity produces impedance followed by recovery and resolution when sufficient time, resources, and reserve are available. Recovery may restore an individualized functional range, support adaptive recalibration, or expand reserve through hormetic remodeling. The framework predicts that such adaptation may produce smaller impedance excursions and faster resolution during subsequent comparable challenges. **(B)** Impedance lock-in. When pressure persists or recovery is incomplete, residual constraint and reduced reserve increase the burden of subsequent challenges and may promote persistent compensation and progressive lock-in. Impedance lock-in denotes persistent residual constraint, reduced reserve, and diminished network reconfigurability; it is descriptive and does not imply a formally demonstrated dynamical attractor. Continued loss of adaptive capacity may culminate in an exhaustion phase with impaired functional integration.

Maladaptation arises when the initiating pressure persists or the time, resources, or reserve required for recovery are insufficient before subsequent challenge. Incomplete recovery leaves residual constraint and reduced reserve, so a comparable challenge begins from an incompletely resolved state and may encounter greater impedance (Figure 2B). With repetition, compensatory responses may become persistent, reducing network reconfigurability while resources remain preferentially allocated toward continued defense rather than restoration (Kelley et al., 2025; Shaulson et al., 2024). We refer to this persistent state as *impedance lock-in*. The term is descriptive and does not imply a formally demonstrated dynamical attractor. Continued contraction of recovery capacity may further reinforce impedance and, in a possible exhaustion phase, contribute to loss of functional integration (Behnke et al., 2026; Tippairote et al., 2025; Tippairote et al., 2026). Persistence alone does not constitute lock-in: durable physiological or network remodeling may remain adaptive when it preserves or expands functional flexibility, reserve, and tolerance of subsequent challenge.

From a Network Physiology perspective, adaptive impedance should involve temporary, task- and context-dependent reconfiguration followed by restoration or adaptive recalibration of flexible network organization (Bartsch et al., 2015; Bashan et al., 2012; Ivanov, 2021). Neither increased nor reduced coupling is intrinsically adaptive: increased coupling may reflect effective coordination or compensatory recruitment, whereas persistent coupling may indicate unresolved demand; reduced coupling may reflect appropriate release of coordination after the initiating exposure has resolved or been contained, or, during exhaustion, loss of physiological integration. Lock-in should therefore be identified not by coupling or decoupling alone, but by persistent or stereotyped reconfiguration together with impaired recovery, residual constraint or hysteresis, reduced network flexibility, altered task-dependent centrality, and incomplete restoration of functional reserve. The distinguishing prediction is whether network interactions remain appropriate to physiological demand, permit impedance to resolve, and restore or expand subsequent adaptive capacity.

## 6 Clinical translation: operationalizing bioenergetic impedance across phenotypes

Chronic or maladaptive bioenergetic impedance is unlikely to produce a single clinical phenotype. Clinical operationalization should distinguish *four analytically distinct domains*: *energetic pressure or demand; throughput or reserve relative to demand; compensatory allocation; and recovery or functional outcomes*. These domains represent causal positions rather than fixed biomarker categories, because the same finding may occupy different roles according to physiological context and temporal stage. They may be assessed using symptoms, functional or physiological measures, biomarkers, and body-composition features, but no individual finding constitutes diagnostic evidence of impedance. Their significance depends on pattern, temporal sequence, relationship to demand, and reversibility. Because similar patterns occur in established conditions involving altered energetic reserve or resource allocation, these observations are not specific to bioenergetic impedance. Inference of chronic or maladaptive impedance should therefore require convergence across these domains, centered on demonstrable demand–capacity constraint and impaired recovery rather than any single manifestation. Exposure-Related Malnutrition (ERM) is presented as an illustrative candidate phenotype for this framework (Box 2).

BOX 2 **Exposure-Related Malnutrition as an illustrative candidate phenotype.**

*Exposure-Related Malnutrition (ERM)* provides a hypothesis-generating candidate phenotype in which sustained metabolic and adaptive demand may exceed available bioenergetic capacity (Tippairote et al., 2025; 2026). In this model, resources are preferentially allocated toward vital functions and the energetic costs of ongoing stress responses, while growth, maintenance, repair, recovery, and other long-term functions become relatively under-supported. ERM is distinct from conventional malnutrition and does not necessarily imply inadequate nutrient or energy intake. Proposed clinical and biochemical manifestations may reflect different stages or consequences of this process, but are nonspecific and should not be treated as diagnostic criteria. Independent empirical validation remains limited; ERM therefore requires an operational case definition and prospective differential validation and should presently be regarded as a candidate phenotype rather than an established manifestation of network-level bioenergetic impedance.

## 7 Testable predictions of the bioenergetic impedance framework

The bioenergetic impedance framework generates several testable predictions for clinical and applied physiology.

*First*, reducing persistent energetic pressure should improve recovery without necessarily increasing nutrient or substrate supply. If inflammation, hypoxia, sleep disruption, environmental exposure, excessive substrate load, pain, or sustained psychosocial stress contribute to persistent energetic pressure, reducing these pressures should improve physiological flexibility and recovery kinetics independently of major changes in energy intake or body weight. This distinguishes an impedance-based prediction from a simple substrate-deficiency model.

*Second*, improving a limiting determinant of conductance, buffering capacity, or recovery capacity should permit greater adaptive throughput. Improvements in oxygen delivery, vascular perfusion, oxidative capacity, nutrient transport, redox buffering, or metabolically active tissue should reduce impedance and improve subsequent challenge tolerance when the targeted factor is itself limiting throughput or recovery capacity.

*Third*, impedance and recovery should be tested sequentially under standardized challenge. Under comparable imposed demand, greater independently measured demand–capacity mismatch should predict slower recovery, greater residual constraint or hysteresis, altered network reconfiguration, and reduced tolerance of a subsequent challenge. If incomplete recovery leaves residual constraint, repeated standardized challenge–recovery cycles should demonstrate accumulating residual constraint and declining recovery capacity. For example, a standardized exercise challenge could test whether independently measured demand–capacity mismatch predicts recovery kinetics, residual constraint, network reconfiguration, and tolerance of a subsequent comparable challenge. In this setting, an adaptive trajectory would show relatively rapid recovery or adaptive recalibration with restored challenge tolerance, whereas a lock-in trajectory would show delayed or incomplete recovery, residual displacement or constraint, and reduced tolerance of a subsequent equivalent challenge.

Construct validity would require demonstrating prospectively that independently measured demand–capacity mismatch predicts recovery kinetics, residual constraint or hysteresis, network reconfiguration, and subsequent adaptive capacity. Deviation from the pre-challenge state should indicate maladaptation only when accompanied by impaired flexibility, incomplete restoration of reserve, or reduced subsequent challenge tolerance; network measures should likewise be interpreted relative to task, physiological state, and functional outcome rather than coupling strength alone.

*Fourth*, beyond construct validity, bioenergetic impedance should demonstrate incremental explanatory or predictive value relative to established measures. These relationships should provide information beyond baseline disease burden, cardiorespiratory fitness, mitochondrial reserve or metabolic flexibility, inflammatory burden, allostatic-load indices, energy availability, sleep and autonomic status, and frailty or functional reserve. If prospectively defined impedance measures simply recapitulate these established measures without improving prediction of recovery dynamics, network reconfiguration, or subsequent challenge tolerance, this would weaken their value as a distinct construct.

Conversely, delayed recovery or persistent multisystem abnormalities alone should not be sufficient to classify a state as high impedance without independently demonstrated constraint relative to demand. Changes in isolated biomarkers without corresponding improvement in recovery dynamics or adaptive capacity should not be interpreted as evidence that impedance has resolved.

## 8 Discussion

Bioenergetic impedance is intended to complement rather than replace existing frameworks of energetic regulation, stress adaptation, and resource allocation. ERP describes the relationship between energetic potential and flux resistance, whereas metabolic flexibility concerns substrate switching, allostasis and allostatic load address adaptive multisystem regulation and its cumulative cost, and resource-allocation models address prioritization under finite energetic capacity (Ames, 2006; Behnke et al., 2026; Kelley et al., 2025; McCann and Ames, 2009; McEwen, 1998; Picard and Murugan, 2025; Shaulson et al., 2024). Among these frameworks, EAS overlaps most directly with bioenergetic impedance: EAS emphasizes regulatory governance and energetic allocation, whereas bioenergetic impedance emphasizes scale-specific constraints in energetic execution and recovery (Schuler et al., 2026).

Its proposed Network Physiology contribution is to relate demand–capacity constraint to the timing, direction, organization, flexibility, and reversibility of cross-system interactions across biological scales, rather than to propose a single mitochondrial or organism-level energetic metric. Whether persistent residual constraint and reduced network reconfigurability correspond to formal attractors in physiological state space remains an empirical question requiring longitudinal analysis and is not assumed by the framework. The conceptual relationships and distinguishing predictions of these frameworks are summarized in Supplementary Table S1.

At present, bioenergetic impedance remains a proposed systems-level construct rather than an established measurable quantity. Evidence for its component mechanisms establishes biological plausibility but does not independently validate the higher-order construct. Its utility will depend on whether prospectively defined demand–capacity constraints predict recovery dynamics, network reconfiguration, and subsequent adaptive capacity, and provide incremental explanatory or predictive value beyond established measures.

## Data Availability

The original contributions presented in the study are included in the article/Supplementary Material, further inquiries can be directed to the corresponding author.
