# Supplementary material for: Bioenergetic impedance: a network physiology framework for stress adaptation, maladaptation, and recovery
Source: Front Netw Physiol. 2026 Sep 29;6:1967461. doi: 10.3389/fnetp.2026.1967461 (PMC13658563; doi:10.3389/fnetp.2026.1967461)
Supplement: Supplementary file 1 [file Table1.docx]

**Supplementary Table S1.** Conceptual positioning of bioenergetic impedance relative to related energetic, stress-adaptation, and resilience frameworks

| **Framework** | **Core proposition / constraint** | **Principal scale and organizing mechanism** | **Adaptive–maladaptive transition, recovery, and empirical emphasis** | **Relationship to bioenergetic impedance** |
| --- | --- | --- | --- | --- |
| **Energy Resistance Principle (ERP)** (Picard & Murugan, 2025) | Energy transformation requires resistance; excessive energetic resistance arises when energy potential exceeds available flux capacity and can generate biological stress. | Fundamental energetic relationship across biological scales; focuses on energy potential, flux, and resistance within biological energy-transforming systems. | Adaptive resistance enables useful energy transformation; excessive/persistent resistance is associated with reductive/oxidative stress, inflammation, damage, and impaired energetic flow. Empirical emphasis is on energetic potential–flux relationships and their biological consequences. | Provides the *potential–flux foundation* for BI. BI extends this logic into time-dependent stress-response and physiological networks by incorporating scale-specific throughput constraints, buffering, compensatory responses, propagation, and recovery. |
| **Metabolic flexibility**  (Muoio, 2014) | Health requires metabolism to adjust fuel selection and oxidative metabolism appropriately to changing substrate availability and energetic demand. | Cellular, tissue, and whole-body metabolism; substrate switching, fuel oxidation, and matching of metabolic pathways to demand. | Flexible switching supports adaptation; persistent inability to match substrate use to conditions constitutes metabolic inflexibility. Typically assessed through metabolic challenges, substrate oxidation, insulin sensitivity, respiratory exchange, or related dynamic measures. | Describes *flexibility of substrate utilization*, whereas BI asks whether total energetic demand can be executed and recovered across multiple conductance processes. Metabolic inflexibility may therefore be a determinant or manifestation of impedance rather than equivalent to it. |
| **Allostasis / allostatic load** (McEwen, 1998; McEwen & Wingfield, 2003) | Stability is maintained through coordinated physiological change; repeated or prolonged adaptation can accumulate physiological cost. | Whole-organism multisystem regulation involving neuroendocrine, autonomic, metabolic, cardiovascular, and immune processes. | Allostatic responses are adaptive when appropriately engaged and resolved; persistent or repeated activation contributes to allostatic load. Empirical emphasis commonly uses multisystem biomarker composites rather than energetic throughput itself. | Provides the broader *adaptation–cost framework*. BI specifies a proposed demand–capacity constraint through which persistent demand relative to throughput and recovery capacity may contribute to incomplete resolution and altered network organization. |
| **Brain–Body Energy Conservation (BEC) model of aging**  (Shaulson et al., 2024) | Cellular aging and damage increase local energetic costs; signals of somatic hypermetabolism are integrated by the brain, which conserves a shrinking organismal energy budget by suppressing lower-priority expenditures. | Aging-focused cell → brain → body architecture; the brain acts as an energetic broker integrating metabolic and stress signals and coordinating systemic conservation. | Conservation can protect energetic balance, but persistent suppression of lower-priority functions contributes to fatigue, inactivity, endocrine/immune alterations, and aging phenotypes. Focuses on energetic costs of aging, systemic signals, brain sensing, and conservation responses. | Strongly overlaps with BI in *bottom-up signaling and systemic energetic reorganization*, but BEC specifically explains aging through brain-mediated conservation. BI is stressor- and exposure-general and focuses on throughput/recovery constraints and dynamic coupling across physiological networks rather than privileging the brain as the central broker. |
| **Nutrient Triage Theory**  (Ames, 2006; McCann & Ames, 2009) | Scarce essential micronutrients are preferentially allocated to functions required for short-term survival and reproduction at the expense of functions supporting long-term health. | Molecular/cellular allocation among micronutrient-dependent proteins and pathways under nutrient scarcity. | Triage preserves immediately essential functions during deficiency, while prolonged inadequacy compromises maintenance functions and may promote age-related disease. | Provides an early *resource-prioritization principle under nutrient scarcity.* BI is broader in both input and scale: nutrient limitation is one possible determinant of impedance among heterogeneous exposures, while BI focuses on constraints in energetic execution, recovery, and propagation through physiological networks. |
|  |  |  |  |  |
| **Energy Constraint on Human Health** (Behnke et al., 2026) | Evolutionary limits on energy transformation and dissipation force an organismal economy of energy and trade-offs among competing processes. | Organelle-to-organism energetic economy; energetic demands are organized into Vital, Stress, and Growth–Maintenance–Repair (GMR) functions, with more urgent functions preferentially supported under constraint. | Temporary trade-offs may preserve survival; persistent energetic competition can divert resources from GMR and impair health over time. Empirical emphasis is on energetic expenditure, compensation, allocation, and trade-off patterns. | Provides a particularly close *finite-capacity and trade-off framework*. BI does not primarily classify where finite energy is spent; it asks how heterogeneous demand encounters constraints in energetic execution and recovery and how unresolved constraints propagate into altered network organization. |
| **Allostatic Triage Model of Psychopathology (ATP Model)**  (Kelley et al., 2025) | Stress can raise energetic demand beyond available production capacity, producing energy scarcity and allostatic triage toward lower-cost functions supporting short-term survival. | Primarily brain/CNS and psychopathology; integrates mitochondrial regulation, allostatic-interoceptive processing, affect-driven prioritization, and redistribution of energy among functional brain networks. | Triage is adaptive during acute scarcity; chronic metabolic inefficiency and affective dysregulation can sustain redistribution from costly executive/long-term optimization networks toward survival-related networks, with persistent patterns becoming canalized. Empirical predictions emphasize metabolic efficiency, affect, stress responses, and CNS functional connectivity. | Shares BI's emphasis on *demand–capacity constraint, network reorganization, and persistence*, but its distinctive target is affect-driven CNS network triage and psychopathogenesis. BI extends across cellular, tissue, organ, and whole-body physiological networks and does not require brain-mediated prioritization or psychopathology. |
| **Energy Allocation System (EAS)** (Schuler et al., 2026) | Resilience depends on coordinated allocation of finite energetic resources under constraints imposed importantly by mitochondrial reserve capacity. | Primarily organismal regulatory governance: SNS together with HPA, HPT, and HPG axes coordinates mobilization, metabolic pacing, conservation, and long-term investment. | Balanced and transient allocation supports resilience; sustained energetic strain promotes mobilization- or conservation-dominant configurations and impaired recovery. Proposed assessment includes mitochondrial reserve/recovery proxies together with coordinated endocrine, metabolic, inflammatory, and functional patterns. | Probably the *closest allocation framework*. EAS principally emphasizes regulation and prioritization of energetic resources; BI emphasizes energetic *execution*—how organism-level demand encounters delivery, substrate-handling, oxidative, redox, buffering, repair, and recovery constraints—and how these constraints feed back into physiological-network organization. |
| **Bioenergetic impedance (BI)** | Impedance arises when energetic pressure exceeds scale-specific capacity for throughput and recovery; it is not synonymous with resistance, mitochondrial dysfunction, inflammation, or any individual mechanism. | Exposure/stressor → whole-organism demand ↔ scale-specific energetic execution. Unresolved local constraints generate compensatory/signaling responses, resource reallocations, and reciprocal propagation through metabolic, immune, autonomic, neuroendocrine, vascular, and behavioral systems, together with nutrient redistribution. | *Adaptive impedance cycling*: transient constraint followed by restoration, adaptive recalibration, or expansion of reserve. *Impedance lock-in*: incomplete recovery, persistent residual constraint, persistent compensation, and reduced network flexibility. Empirical emphasis is on dynamic challenge–recovery paradigms, scale-specific throughput/recovery measures, and cross-system coupling/topology. | Proposed distinctive contribution: connects heterogeneous exposures and organismal demand to energetic execution and reciprocal Network Physiology dynamics. It predicts that persistent impedance should be associated with persistent or stereotyped network reconfiguration, altered task-dependent coupling or centrality, reduced network flexibility, and incomplete restoration of functionally appropriate organization and adaptive capacity. These relationships should provide incremental information beyond established measures of reserve, allocation, metabolic flexibility, or cumulative physiological burden |

**Note:** The frameworks are presented as complementary rather than mutually exclusive. Domains are consolidated for conceptual comparison; differences reflect primary explanatory emphasis rather than exclusive biological scope. Bioenergetic impedance is proposed as a scale-dependent Network Physiology construct and not as a universal quantitative measure or a replacement for the frameworks summarized above.
